# Investigating Sport Stakeholders’ Understanding of Behaviour Management within a Competitive Youth Baseball Team

**DOI:** 10.3390/sports11030069

**Published:** 2023-03-17

**Authors:** Joseph John Gurgis, Gretchen Kerr, Anthony Battaglia

**Affiliations:** Faculty of Kinesiology and Physical Education, University of Toronto, Toronto, ON M5S 2W6, Canada

**Keywords:** behaviour management, exercise, benching, yelling

## Abstract

The following study employed an instrumental case study to investigate sport stakeholders’ understandings of behavioural management strategies used in competitive youth baseball, including the identification of common strategies and interpretations of these as punishment or discipline. Twenty-one participants, from one competitive (AAA) all-boys baseball team, including three coaches, eleven baseball players, and seven parents, were recruited to participate in an individual semi-structured interview. Interviews ranged between 30 and 150 min, and data were analyzed using reflexive thematic analysis. Several behaviour management tactics were identified, of which exercise, benching and yelling negative comments were most often reported. While participants interpreted excessive exercise and benching as punitive and/or disciplinary approaches to behavioural management, yelling was consistently viewed as punitive. Participants confused punishment and discipline as interchangeable, thus suggesting a lack of awareness regarding developmentally appropriate strategies of behavioural management and highlighting the normalization of certain punitive tactics in youth sport. The results underscore the necessity of imparting knowledge to the sports community regarding age-appropriate behavioural management interventions to foster safe and enjoyable athletic experiences for youth competitors.

## 1. Introduction

When designed and delivered in a developmentally appropriate manner, participation in organized sport has been linked with positive physical and psychosocial outcomes for youth [1,2]. Informed by sport-specific literature [3], the term ‘youth’ is used to broadly refer to participants between 10 to 18 years. Specifically, participation in organized sport can enhance self-esteem, improve social skills, increase confidence, reduce depressive symptoms [4] and improve overall health behaviours among youth, such as better eating habits, decreased drug use, and safer sexual practices [5]. The attainment of positive developmental outcomes is significantly influenced by youth athletes’ experiences with critical socializing agents, such as coaches and parents [6]. In fact, Dorsch et al. [7], referred to coaches and parents as vital “gears” who are seen as the most proximal and influential stakeholders to athletes within youth sport. One plausible avenue for these socializing agents to impact the development of young athletes is through the utilization of behaviour management techniques in sport, including punishment and discipline.

Punishment is defined as the application or withdrawal of a stimulus by an authority figure in response to perceived wrongful behaviour, which is meant to decrease the likelihood of that behaviour being repeated [8]. Punishment manifests through positive and negative forms; positive punishment occurs through the direct application of an aversive stimulus (e.g., spanking), whereas negative punishment is characterized by the removal of a rewarding stimulus (e.g., timeout) [9]. In the parenting and education literature, the negative consequences of punishing youth are well-documented and include impaired adult–child relationships, decreased abilities to internalize moral values, lower self-esteem, antisocial behaviour, and the normalization of physical aggression as a strategy for solving conflicts [10,11,12]. 

Informed by the detrimental effects of punishment, a shift from using punitive practices to developmentally appropriate disciplinary strategies has occurred in parenting and education [13,14,15]. Discipline is understood as a process of nurturing and teaching that facilitates the development of self-control, competence, and self-direction among children and youth [16]. Disciplinary strategies include, but are not limited to, engagement and reasoning, goal setting, and rewarding appropriate behaviour [16,17] and have been associated with benefits such as improved self-esteem, enhanced relationships, empathy, a greater ability to regulate emotions and distinguish right from wrong [18].

Despite the adoption of more positive disciplinary approaches in other child-populated domains, research on behaviour management in sport suggests that punishment continues to be used to modify athlete behaviour. For example, researchers have examined common forms of punishment used in sport, such as excessive exercise (e.g., push-ups or sprints until exhaustion), yelling, and benching (i.e., removal of playing time) as consequences of perceived undesirable behaviour (e.g., poor attitude, arriving late, and inadequate performances) [19,20,21,22]. Collectively, these punitive methods have been associated with negative outcomes for youth athletes, which include but are not limited to fatigue, injuries, negative perceptions of the self, tarnished sport relationships, impaired learning, and a lack of desire to continue playing [19,20,23].

In response to documented negative repercussions of punishment use, researchers, sport leaders and policymakers have denounced the use of coaching tactics such as excessive exercise and yelling [24,25,26], and instead, have advocated for more humanistic approaches to behaviour management. For example, Harris-Reeves et al. [17] suggested establishing and explaining rules and/or expectations to athletes, communicating unacceptable behaviours and associated consequences, using reminders throughout practices, positive verbal communication and rewards, and offering athletes choices. Despite the calls for alternative approaches to youth behaviour management in sport, the use of punishment in sport remains a pervasive strategy used by physical education teachers, sport coaches, parents and parent-coaches to modify perceived misbehaviour [21,27,28,29].

Based upon the evidence of harms associated with punishment use, and the shift from the use of punitive to disciplinary strategies in other youth-populated domains, a question is raised about why punishment continues to be used in sport contexts. While researchers have examined the perceived reasons for use and effects of behavioural management tactics in youth sport [19,20,30], to-date, no research has investigated how stakeholders understand and interpret punishment and discipline, and whether common behavioural management tactics are viewed from punitive or disciplinary lenses. Understanding stakeholders’ interpretations of behavioural management strategies is important to prevent harms that may result from punishment use and to better understand where interventions are needed to advance the use of developmentally appropriate disciplinary strategies. As such, the current study investigated sport stakeholders’ understandings of behaviour management strategies used in youth sport. Specifically, we were interested in sport stakeholders’ identification of common behavioural management strategies used in youth sport and their interpretations of these practices as disciplinary and/or punitive in nature.

## 2. Materials and Methods

The current research employed an instrumental case study approach to investigate the conceptualizations of behaviour management among athletes, coaches, and parents of a competitive youth baseball team at the AAA level [31]. The goal of an instrumental case study is to gather insights about a specific case or phenomenon to understand its unique details and how it functions within various contexts or to redraw generalizations of the phenomenon [31,32]. In this study, the focus was on behaviour management in the context of competitive youth baseball, including how it is understood, experienced, and implemented. The youth sport context was chosen as early sport experiences (e.g., behavioural management) determine the nature and quality of subsequent sport experiences and, thus, the extent to which social, emotional, and cognitive development is fostered [1,2]. Further, the choice of competitive baseball was motivated by the sport’s distinctive normative and well-documented acceptance of punitive practices, specifically at more competitive levels [33].

### 2.1. Research Paradigm

The current study is situated within a constructivist paradigm, which permits us, as researchers, to critically discuss and reflect on varying conceptions of behaviour management in sport with our participants [32,34]. Constructivism embraces a relativist ontology, which assumes experientially and socially constructed realities exist as mental constructions [34]. The ontological assumption of this study suggests varying interpretations—or constructions—of behaviour management may exist between participants. A constructivist paradigm further embraces a transactional, subjectivist epistemology [34], which would perceive the participant(s) and researcher(s) as being interactively connected insofar that findings are developed as the research proceeds [34]. The epistemological assumption of this study required the researchers to acknowledge how their formal education in the field of sport psychology and experiences as former athletes, coach educators, and mental performance consultants, may shape their perspectives regarding punishment and discipline in sport. Specifically, given the principal investigator’s previous experience investigating coaching practices in sport (e.g., [35]), and his role in conducting the interviews, it was important for him to reflect on how his research and sport experiences influenced the parameters among which he and the participants discussed punishment and discipline, behavioural management strategies in sport and the co-created themes. This process of self-reflection was achieved through maintaining journal notes on member reflections and conversations with the co-authors who served as critical friends [36].

### 2.2. Case/Team Description

The case of interest was a competitive all-boys baseball team from a large city in Canada. According to the league the team was affiliated with, the team is classified as a 14U Triple A (AAA) youth baseball team, which is considered one of the highest levels of competition for baseball participants within this age group. There were twelve boys affiliated with the team, three male coaches, and twenty-two parents/caregivers. The season was eighteen weeks long and consisted of three preseason games, thirty in-season games (fifteen home games and fifteen away games), three local tournaments, and one international tournament. Collectively, the boys practiced between two and three times per week. The team was affiliated with an organization that the parents described as notorious for selecting “intense and unorthodox coaches”. The principal investigator, who coached and umpired in this league, had witnessed firsthand the wrath of such coaches (e.g., yelling at parents and kids, throwing equipment, being thrown out of games). To be included in this study, participants had to be affiliated with this team in the capacity of an athlete, coach, or parent/caregiver.

### 2.3. Participants

Case studies are typically described as being specific and bounded in place and time [32]. Thus, participants were purposefully recruited from one competitive baseball team in a large city in Canada. The principal investigator initially contacted the head coach via email; contact information was retrieved through the publicly accessible baseball club website. Upon coach approval, the principal investigator was invited to a team meeting to discuss the study with the assistant coaches, parents, and athletes. To be included in this study, participants had to be affiliated with this team in the capacity of an athlete, coach, or parent/caregiver. Recruiting participants from the same team was intended to improve the likelihood that the sample would share similarities associated with the research questions [37] and permitted the research team to gather greater insights about the case of interest.

Case study research is less concerned about answering questions of ‘how much’ or ‘how many’ but rather focuses on answering questions of ‘how’ and ‘why’ [32]. As such, the research team prioritized the intricate complexities of this team as a case [32]. Participants of this study included three youth baseball coaches (one head coach and two assistant coaches) (Ages: 45–50 years), eleven youth baseball players (Ages: 13–14 years), and seven parents (Ages: 40–50 years), all affiliated with the same competitive (AAA) baseball team. The three coaches identified as men; the head coach reported twenty years of baseball coaching experience across different competitive levels and had previous experience as an inter-university baseball player. The two assistant coaches reported 3–5 years of coaching experience and had no prior experience playing baseball. All the athletes identified as boys, possessed 5–7 years of playing experience, and participated in 10–14 h of baseball per week, between dryland training, practices, and games. Four of the parent participants identified as men and three as women. Of the eleven athletes, seven represented athlete-parent dyads and four participated without their parents.

Ethical approval for this study was obtained from the University Research Ethics Board (Ethical Issue Number: 31648). Coaches and parents were asked to provide written consent prior to participating, whereas athletes were required to provide written assent, given they were under the age of consent (i.e., 16 years). Additionally, written consent from the parents was elicited to confirm they approved of their child’s participation in the study. To maintain participant confidentiality, a pseudonym was assigned to each participant, and all personal identifiers were removed. Youth athletes’ perspectives were sought given previous research which has suggested punitive strategies may have a detrimental impact on developmental outcomes (e.g., diminished fun) [19,20]. Furthermore, athletes’ perspectives must be considered if stakeholders truly wish to formulate and/or revise policies and programmes concerning behaviour management that are developmentally appropriate [38]. Finally, given the influential role of coaches and parents in shaping the nature and quality of youth’s sport experiences [39,40], it was imperative to include their perspectives on behavioural management.

### 2.4. Data Collection

Consistent with the constructivist paradigmatic position upheld for the research, participants were invited to share their perceptions of behaviour management strategies used in youth sport and asked to describe whether they perceived these strategies as punishment or discipline. One individual, face-to-face, semi-structured interview was conducted with each participant. The length of the interview varied across the three participant samples; athlete interviews ranged between 30 and 65 min, parent interviews ranged between 70 and 110 min, and coach interviews ranged between 90 and 150 min. The principal investigator conducted all interviews, which were transcribed verbatim and, with the permission of the participants, were audio-recorded.

The interviews began with broad, open-ended introductory questions, pertaining to behaviour management. For example, coaches and parents were asked questions, such as “What are common tactics used to manage athlete and/or team behaviours on your team?” Similarly, athletes were asked, “What happens when you or your teammates do something wrong, such as arrive late to practices/games, not pay attention, or disrespect others?” After the participants’ descriptions of the common methods used, all participants were asked whether they understood these methods as punishment or discipline and probed to explain why. Probes were intentionally used to clarify participants’ responses and to gain a more comprehensive understanding of participants’ interpretations of behaviour management strategies used in youth sport. The findings contained in the current study focus specifically on participants’ interpretations of behaviour management strategies used in youth sport as forms of punishment or discipline.

### 2.5. Data Analysis

Interview transcripts were analyzed using a reflexive thematic analysis (TA), characterized as an open and recursive process used to provide clear and convincing interpretations of the data [41]. The reflexive nature of this analytic approach aligns with our epistemological position that assumes knowledge is produced from the reflection, transaction, and negotiation of information [42]. As an example of theme development using a reflexive thematic analysis approach, following interview transcriptions, the principal investigator immersed himself in the datasets to become familiar with participants’ interpretations, beliefs, and explanations related to behaviour management in sport. In this phase, he regularly read through transcripts, wrote memos, engaged in critical conversations with his co-authors to compare interpretations, and sought connections between the datasets. Next, succinct codes were inductively generated to aid in identifying larger segments of data. Several semantic, surface-level codes were identified in this phase, including: “interpretations of behaviour management”, “bad behaviours”, “exercise, benching, and yelling negative comments as behaviour management”, and “punishment and discipline”. Then, themes were constructed, revised, and defined, respectively. Many of the initial semantic codes identified were grouped based on perceived similarities to form preliminary themes. For example, the codes “interpretations of behaviour management” and “exercise, benching, and yelling negative comments as behaviour management” were combined to construct the theme “Methods of Managing Athlete and Team Behaviours”. Additional themes, such as “Normative Behavioural Management Strategies Used in Sport” and the associated subthemes, as well “Conceptual Confusion around Punishment and Discipline”, were generated following ongoing discussions among the research team and with participants. The final phase required us to generate a final report; multiple drafts were developed and circulated among the research team for revisions.

A collaborative approach to data analysis was adopted for the current study. For example, following interview transcription, the principal investigator engaged in member reflections with participants; this was to ensure the passage of time did not negatively impact the participants’ ability to recall information discussed throughout the interview. Member reflections facilitated critical dialogues, which led to a better understanding of participants’ views, the generation of new insights, clarification of thematic interpretations, and the development of new supporting evidence [36]. For example, throughout the interviews, participants often referred to instances of punishment and discipline; however, seeking clarifications after interview completion helped the principal investigator to understand that stakeholders were, in fact, using the terms synonymously and were often unaware that these terms were distinct. Member reflections also enabled a deeper understanding of participants’ rudimentary interpretations of discipline as positive and punishment as negative.

Further, the co-authors, who served in the capacities of a supervisor and colleague, operated as critical friends throughout the data analysis process. Specifically, research team members routinely engaged in collaborative discussions throughout the data analysis process to reflect on and discuss emerging themes and to ensure the participants’ views and main messages of the data were adequately represented [36]. These discussions were characterized by the sharing and deliberation of pertinent literature and the use of provocative questions related to behaviour management that aided in the identification and organization of themes [43]. Informed by the collaborative approach to data analysis, the researchers made an interpretive judgement regarding the overarching themes presented and discussed throughout the paper.

## 3. Results

The purpose of this study was to investigate sport stakeholders’ understandings of behaviour management strategies used in youth sport, including the identification of commonly used strategies and interpretation of these strategies as discipline and/or punishment. Participant responses revealed that exercise, benching, and yelling negative comments were the most common behaviour management strategies used with this youth sport team. Participants struggled to differentiate exercise and benching as punitive or disciplinary in nature but consistently interpreted yelling negative comments as punitive. The responses presented are not unique to each athlete, parent, and coach but represent overarching interpretations and common meanings identified throughout all participants’ responses.

### 3.1. Methods of Managing Athlete and Team Behaviours

The participants identified exercise, benching, and yelling as the most common behavioural management strategy used by coaches. Some participants referred to setting up the field, cleaning up equipment after practices and games, and in extreme situations, asking an athlete to stay home as additional strategies to manage athlete and team behaviour.

#### 3.1.1. Exercise

All participants referred to the use of exercise (e.g., push-ups, running laps, or running wind sprints) as a method of correcting athlete behaviour perceived as undesirable or problematic. Mina (athlete) shared, “It’s not uncommon for us to run after a bad loss or practice”. Patrick (assistant coach) attested to the use of exercise in response to poor performance, as he stated, “if they [team] don’t want to work hard when it counts, then they’re going to stay back and work hard after. Usually, a few wind sprints reminds them to come to the field ready to work”. Exercise was also frequently used to address behaviours perceived as disrespectful. For example, Laura (parent) explained, “kids would run laps if they were disrespectful. Swearing at a coach, a player, an umpire…if there’s disrespect, then there’s going to be kids running laps…they’re going to feel some pain…they’d run until they’re exhausted”.

#### 3.1.2. Benching

Many participants identified benching as a behaviour management strategy that coaches used to manage individual athlete behaviours. For example, Derrick (assistant coach) admitted, “We [coaches] bench players based on their performance. I’d be the first to say, ‘bench my son’ if he’s playing like crap”. Diego (athlete) agreed that benching was used as a response to individual poor performance: “We’re playing high level baseball. If you’re not playing good, you’re sitting. That’s only fair. It’s not great, but you can only play nine guys. The nine best should play, while the others sit until they prove otherwise”. Similar to exercise, participants indicated benching could be used in response to other undesirable behaviours, such as arriving late to practices or games. Phil (parent) stated, “Showing up late to practice or worse, a game, means you don’t care. So, sit on the bench. If you don’t care about your team or coaches enough to show up on time, then sit out”.

#### 3.1.3. Yelling Negative Comments

The participants recognized yelling negative comments as a form of behaviour management used by coaches when they were extremely upset. For example, Eric (athlete) shared, “…we played like absolute shit, I think we lost by 8 or 9, and [coach] laid it on us…he told us we’re wasting his time. He said we’re so bad that we’d strike out playing t-ball”. Alyssa (parent) explained that coaches would yell in response to aggressive behaviours such as fighting: “The coaches expect the boys to respect one another. When they start fighting, either physically or verbally, you can expect the coaches to yell. I know that type of behaviour makes them so mad”. Finally, Patrick (assistant coach) admitted, “if they [athletes] give me lip, they’re going to get it back tenfold. I’m not volunteering my time to be disrespected”.

#### 3.1.4. Setting up the Field, Cleaning up Equipment, and Asking Athletes to Stay Home

Although not as frequently referred to, the participants identified alternative behaviour management strategies in addition to exercise, benching and yelling. Following a bad game, Jim (head coach) revealed he has asked the athletes to set up the field and clean up the equipment:

I like to get to practice early to clear the tarps and rake the infield. Sometimes I even give the grass a quick cut. It would take me about an hour to do everything, then we practice for two hours, and then clean-up for another half hour. If the boys have a bad game, then next practice, they’re on set-up-clean-up duty. On those days, I make sure we cut the grass.

In one extreme example, Martin (athlete) admitted he was instructed to stay home from practice for purposefully attempting to injure an opponent during a game. Martin shared, “I was pitching, and I meant to hit the [batter] in the back but I hit his head. Thankfully, he had a helmet. Coach pulled me and told me not to come to next practice”.

### 3.2. Normative Behavioural Management Strategies Used in Sport

As previously discussed, the participants cited exercise, benching, and yelling negative comments as the most common behavioural management strategies used on this sport team. When probed about these tactics, many participants struggled to determine if exercise and benching were used punitively or disciplinarily, but there was a consensus amongst the group that yelling negative comments was used punitively.

#### 3.2.1. Exercise as a Punitive and Disciplinary Method

According to the participants, the use of exercise was a common tactic used to manage youth athletes’ behaviours. Many participants understood exercise as a strategy that can be used to punish and discipline athletes whose behaviour was perceived to be disrespectful, unfocused, and lazy. Derrick (assistant coach) explained “exercise as punishment is directly correlated to disrespectful behaviour towards your coaches, teammates, officials. Exercise as discipline is about a lack of effort, being lazy on the field, careless mistakes. These are unacceptable and exercise gets them to refocus”. Eric (athlete) also explained that exercise was a commonly used punishment in response to disrespectful behaviour but suggested that if you learn from the punishment, it then constitutes discipline: “If you are being disrespectful to the coach, then obviously exercise is being used as punishment because you shouldn’t be disrespecting someone who is volunteering their time…if you learn your lesson from the exercise, then it’s discipline”.

Laura (parent) shared similar sentiments about exercise being both punishment and discipline: “[Exercise] is punishment because they are being punished for bad behaviour, but it’s discipline because they are learning to take responsibility for their actions…they learn from the pain which teaches them not to screw up again”.

Feelings of pain associated with excessive exercise were reportedly necessary for athletes to learn right from wrong through exercise. Val (athlete) explained, “when you feel pain it’s punishment because it’s meant to hurt you. It tells you not to screw up again or else this is what’s going to happen…nobody wants to experience pain”. Additionally, Phil (parent) stated, “it’s gotta hurt at least a little bit. It’s what makes exercise an effective punishment”. In contrast, participants such as Mina (athlete) referred to experiences of pain as an indicator of an effective disciplinary strategy: “Twenty push-ups or five laps every time I screw up, well now it’s going to hurt a bit. That’s when you learn what not to do, so [exercise] would be discipline when it’s painful because we’re learning”.

When referring to the use of exercise, the participants also acknowledged the intensity of the activity as a potential factor in distinguishing the experience as punishment or discipline. For example, Tim said, “…punishment is more of you being bad so now you have to do ten laps. Discipline, I feel like, is running four laps instead of ten”. Similarly, Caleb (athlete) stated:

If there’s a visible difference, like…doing ten laps and knowing your players can’t do it versus doing one lap and having a conversation with the player about what he did wrong and how he could improve, that’s the difference between punishing and disciplining.

Although some athletes perceived ten laps as severe punishment, Patrick (assistant coach) perceived ten laps to be a reasonable form of discipline: “…ten laps isn’t that bad…maybe twenty would be punishment”. When asked to explain the most effective way of managing misbehaviour, Patrick responded, “Run them until it hurts. I don’t think you need to do it all the time, but sometimes I think it helps. Gets them focused and paying attention to us”. Derrick (assistant coach) agreed that running was an effective method of managing behaviour because “it teaches them [athletes] to wake up…that the mistakes they have made are unacceptable and that they are better…a good hard run gets the job done”. Overall, the quotations contained within this section highlight conceptual confusion regarding the use of exercise as a behaviour management strategy, with participants suggesting exercise is used as punishment, discipline, or both.

#### 3.2.2. Benching as a Punitive and Disciplinary Method

Similar to exercise, benching as a behavioural management strategy was perceived as both a punitive and disciplinary method. However, unlike stakeholders’ perspectives of exercise, views of benching differed between stakeholders with athletes viewing benching as punitive, and adult participants predominantly suggesting it was disciplinary. For example, Diego (athlete) suggested, “[benching] is punishment…not playing someone is the worst thing you can do to an athlete. It’s an extreme punishment”. Likewise, Martin (athlete) stated, “benching is the worst punishment. If I make a mistake and coach sits me, he’s punishing me for not being good enough. It’s a terrible feeling”. Alyssa (parent) agreed that benching is used in response to poor performance; however, she suggested it was a disciplinary tactic:

Baseball is a team sport and if they’re not playing well, then they need to sit. Having them out there does no good to them or the team. If a player keeps on making the same mistake, he’s just going to feel bad about himself and feel bad about hurting the team, so you have to sit him…[benching] is purely disciplinary. It teaches kids the importance of sacrifice, putting the team’s needs above your own. These boys aren’t babies. This isn’t t-ball anymore. These are elite boys who can learn a lot from the sidelines.

Alyssa’s comment suggests benching may teach values such as sacrifice and selflessness, which can benefit the team, thus making it an appropriate disciplinary strategy to be used at the competitive level of sport. The benefits of benching as a disciplinary strategy were echoed by Derrick (assistant coach), who shared:

I use benching as discipline. It’s good…because it’s quick feedback. For me…the best form of feedback…I’m okay with benching. I’m trying to convey expectations through benching, trying to build character and resilience. I want the boys to know they need to work hard and earn their spot on the field…benching isn’t reactive like punishment. It’s a strategic form of discipline.

Participants noted that the amount of time on the bench influenced whether they interpreted benching as punishment or discipline, suggesting that benching for a short period of time constituted discipline whereas longer periods of time spent sitting out was punishment. Brian (athlete) clarified:

I guess it depends on how many innings you sit. If you make a bad throw to home from right field, discipline would be maybe, sit an inning, reflect on what you did…if you make an error and strike out a bunch of times and bring your team down, maybe sitting the whole game then would be punishment.

Erica (parent) agreed that extended periods of time spent on the bench represented punishment: “To punish individuals you can bench them the whole time…make them sit out a whole game”. Similarly, Patrick (assistant coach) stated, “If you sit a kid an entire game, maybe back-to-back games, that’s punishment. A few innings each game, or for one game, then that’s discipline. It’s not going to break them. It can actually benefit them”.

#### 3.2.3. Yelling Negative Comments as a Punitive Method

Unlike the use of exercise and benching, yelling in the form of critical, demeaning, or humiliating comments, was interpreted as a punitive method by all participants. Parents, for example, agreed that yelling was the worst method of punishment for an athlete to endure, leading to feelings of shame and embarrassment. Erica (parent) suggested, “yelling is probably the worst punishment the kids can experience on the field. No one ever wants to be yelled at…it’s such a shameful punishment. And it doesn’t work”. Further, Alyssa (parent) shared:

Yelling is pointless…yelling is just an expression of your frustrations…[Son] would rather die than be yelled at by a coach. He just hates it. He’d be mortified to have a coach call him out in front of his friends. He’d quit baseball.

The athletes agreed that yelling was a form of punishment and was a harmful and ineffective method of changing behaviour. Karim (athlete) compared the ineffectiveness of yelling at athletes to when he yells at his dog: “When I yell at my dog for barking, he’ll bark even more. Kids are the same. They won’t respond well”. James (athlete) expressed similar beliefs, suggesting that yelling may deter athletes from participating in sport: “Yelling doesn’t help, it just discourages people…That kind of punishment can scare kids away”.

Yelling was perceived as ineffective due the potential negative outcomes associated with using this tactic. For example, Jim (head coach) stated, “Yelling is pointless. It serves absolutely no purpose other than discouraging kids. They may hate you. They may leave the team or the sport…yelling demonstrates an inability to communicate and lead, which we’re called to do as coaches”. In addition to producing feelings of animosity, yelling was perceived as eliciting fear of failure and anxiety, thus negatively impacting performance. Tim (athlete) commented:

There’s already so much thinking in baseball and when I’m in a slump, I can’t help but think of the shit I’m going to get if I make a mistake. Sometimes I like when coach sits me in those situations because I’m afraid of getting yelled at if I make a mistake. I don’t want to sit, but I’m also anxious of getting hell after…I just hate getting yelled at.

Laura (parent) stated, “yelling at them [athletes], makes it difficult for them to find the courage and drive to fight through the errors and the losses…it’s a bad type of punishment that can really affect the team”. Likewise, Phil (parent) shared:

No, kids are not supposed to get yelled at because when you yell at the kids, what do you think is going through that kid’s mind? The next ball that goes to him he’s going to be so nervous that he’s going to try not to mess up, and that’s when he’ll mess up…if you yell at him, he’s going to go…have you seen a turtle? Right in his shell.

### 3.3. Conceptual Confusion around Punishment and Discipline

Stemming from the stakeholders’ perspectives of the three common behaviour management strategies, and the apparent inability to distinguish between these as punitive and disciplinary, the participants were asked more generally about their conceptualizations of punishment and discipline. According to the athletes, parents, and coaches, punishment and discipline were interpreted as interchangeable; specifically, their responses highlighted the assumption that both approaches achieve similar ends related to behaviour change. For example, Diego (athlete) stated, “There’s not much of a difference…the goal is to change behaviour…punishment, discipline, it’s all the same at the end”. Eric (athlete) echoed similar sentiments, “I see it [punishment and discipline] as the same thing…I don’t see a difference. Both achieve the same thing…both are used to correct behaviour”.

Many of the adult participants (coaches and parents) also suggested that punishment and discipline were similar concepts. For example, Patrick (assistant coach) indicated, “There’s no difference…Punishment, discipline, repercussion, consequence…it’s about stopping bad behaviour. If you can use a strategy that stops athletes from misbehaving, then it shouldn’t matter what you call it, as long as it stops the behaviour”. Similar to the athletes’ responses, parents associated punishment and discipline with desired outcomes (i.e., changes in behaviour). Phil (parent) stated, “they’re more or less the same to me…we’re getting picky about semantics now… Don’t they both do the same thing?” Likewise, Laura (parent) elaborated:

Discipline is a branch of punishment. [Discipline] sounds nicer. It’s probably more politically correct to saying your disciplining a child than punishing. But it’s the same thing. Punishment sounds harsh, like you’re really cracking the whip. But don’t they both do the same thing? If I punish or discipline my child, the goal is still for them to learn right from wrong…so, it’s the same thing. They both work.

Overall, the perspective that punishment and discipline were synonymous concepts was influenced by the participants’ belief that both methods achieve the intended outcome of behavioural change.

In a few instances, participants claimed that punishment and discipline were different, but a very rudimentary distinction was provided, namely, that punishment was negative and discipline was positive. For example, Erica (parent) noted, “…discipline is to teach them a lesson…teach them the right way or teach them the way they shouldn’t be doing it…punishment is a harsher, more negative way of trying fix behaviours”. Beyond this superficial distinction, no substantive differences were conveyed.

## 4. Discussion

The current study investigated sport stakeholders’ understanding of behaviour management strategies within one competitive all-boys youth baseball team, including the identification of commonly used strategies and interpretation of these tactics as punishment or discipline. The findings in the current study provide insight about sport stakeholders’ views of punishment and discipline; of particular interest, the conceptual confusion of punishment and discipline highlights the need to educate coaches and other sport stakeholders on the importance of using developmentally appropriate disciplinary strategies at the youth sport level. Currently, there is a gap in Canada’s coach education system confronting the punitive, yet often normalized use of exercise, benching, and yelling. Increasing sport stakeholders’ (e.g., coaches, parents, athletes) awareness of behaviour management in sport may safeguard vulnerable participants (i.e., athletes) from maltreatment inflicted by harsh punishments.

Across the stakeholder groups, responses highlighted that among several reported behaviour management techniques used on this baseball team, exercise, benching and yelling negative comments were the most common. All stakeholder groups believed that the administration of exercise in response to undesirable athlete/team behaviour was effective in obtaining athletes’ attention, teaching athletes that their behaviour was inappropriate, fostering mental toughness, establishing coach authority, and increasing youth’s ability to distinguish right from wrong, notions which have also been supported by research examining teachers’, teacher-coaches’, physical education majors’, and athletes’ perspectives on the use of exercise as a behavioural management method (Battaglia et al., 2018; Kerr et al., 2020). However, these findings contrast with previous research that indicates exercise as punishment is associated with detrimental effects such as extreme fatigue, injury, impaired coach–athlete relationships, and negative perceptions of self [20,23], and researchers have proposed that exercise as punishment may escalate to a point of constituting psychological or non-contact physical maltreatment [44,45]. Moreover, several physical activity and sport-related organizations have denounced the use of excessive exercise to correct behaviours, highlighting short- and long-term negative effects (e.g., [26]).

For benching, different interpretations existed among stakeholder groups. Coach and parent responses suggest that benching can be an effective behavioural management tactic to address poor performance, build character, stimulate reflection, and encourage values, such as perseverance, sacrifice, and selflessness. Alternatively, athletes viewed this tactic as a form of punishment and one of the worst things a coach can do to an athlete. This finding is not surprising when considering playing time is one of the primary ways youth experience fun and enjoyment in sport [46]. Further, the athletes in the current study suggested that being benched conveyed their lack of worth/skill, supporting previous researchers who have reported that youth athletes do not view benching as an effective behavioural management tool but instead a punishment that has detrimental impacts for their sense of self and worth [20,46,47]. To-date, coaches’ and parents’ perspectives on the use of benching have not been explored. However, comparisons may be drawn between benching and the use of time-out, a practice addressed in the parenting and education literature, characterized by the confinement of individuals, or time spent away from a positively reinforcing environment, for unacceptable behaviours [48]. While the practice of time-out may be beneficial for cooling emotions, it often has deleterious effects on children, sending messages that children are not worthy of the adult’s attention [49,50]. Instead, some authors (e.g., [49]) recommend time-in parenting strategies in which parents provide a misbehaving child additional rather than less attention. The time-in approach conveys to children that parents are available for support and that they believe the child can learn to correct their behaviour on their own; providing children this sense of autonomy is shown to improve a child’s confidence and sense of self [49]. These findings suggest that to reduce the potential negative effects of benching in youth athletes, coaches should provide attention to the athlete through explanations for the benching and recommendations for optimal behaviour once the athlete returns to the field of play. The apparent disconnect between parents’ and coaches’ views of benching and how this practice is interpreted by athletes highlights the need for further research to understand how benching may be implemented in a more developmentally appropriate and strategic manner to foster learning.

In contrast to the findings pertaining to exercise and benching as punishment in which varying perspectives emerged amongst the stakeholders, all groups agreed that yelling negative comments was a form of punishment and had detrimental effects. The stakeholders reportedly viewed this tactic as an inadequate and harmful form of communication that may elicit feelings of low self-worth, fear, and stress. This finding is consistent with previous research that identifies yelling negative comments as a form of emotional abuse in sport [45], which is associated with negative outcomes, such as stress, impaired relationships, and negative perceptions of self [47,51].

There are several reasons to explain why the practices of benching and exercise may be normalized in sport, while yelling is not. It is possible that the use of exercise as punishment in sport continues as a common practice despite documented negative effects for athletes and despite position statements condemning its use because of its long-standing history of use in sport and its military roots [26,52]. The use of exercise as punishment is also a practice unique to environments in which individuals engage in instructional activities related to physical movement such as sport and physical education and thus may be less impacted by normative practices outside of sport and physical education. The struggle of navigating between vigorous, yet beneficial physical exercise, and the punitive, and at times, abusive use of exercise, poses significant challenges that support the advancement of clear definitions [53].

The practices used in competitive youth sport are undoubtedly influenced by the prioritization of outcomes such as performance excellence, including winning-at-all-costs approaches. Several researchers highlight the implicit and explicit cultures of control that exist in organized sport [54], which provide a context in which potentially harmful practices become normalized and reinforced in the pursuit of winning and success [55]. Moreover, the controlling power held by the coach is often recognized as influencing the normalisation of questionable practices in sport, such as the use of punishment [23]. The normalized use of harmful practices in sport, such as exercise or benching as punishment, maybe a consequence of the autonomous nature of organized sport [56,57]. As Bruyninckx [56] claims, sports occur in a separate, autonomous sphere seemingly disconnected from the normative rules and regulations of society. The autonomous nature of many sport organizations has reportedly interfered with responsibility to uphold human rights [57]; consequently, athletes are exposed to harmful practices that often go unquestioned. The acceptance of exercise and benching as effective strategies by coaches and parents in the current study demonstrates a lack of awareness of the negative implications of these practices. Additionally, the well-documented power held by coaches [58] often contributes to parental compliance and support of harmful practices, further placing athletes in vulnerable situations [59,60]. The consistent findings with respect to yelling negative comments, in contrast to benching and exercise, may result from the fact that yelling is generally viewed as unacceptable in sectors outside of sport. In other words, yelling is not acceptable in educational and workplace settings [61], and this generalized view may extend into sport. Benching and exercise, however, are behaviours unique to the sport environment and thus may not be influenced by non-sport related norms.

The results highlight a lack of understanding regarding punishment and discipline. Specifically, the participants failed to distinguish these concepts when interpreting the use of exercise and benching in sport, but instead suggested that these behavioural management strategies can be used as both punishment and discipline. In the few instances where participants acknowledged punishment and discipline as different, these interpretations did not expand beyond the general understanding that punishment was negative and discipline was positive. Some of the participants referred to conditions such as the length of time an athlete was benched, the duration and intensity of exercise administered, and whether pain was experienced to distinguish between punishment and discipline. Further research is needed to explore the conditions under which discipline is distinguished from punishment.

In several interpretations, participants distinguished a tactic as punitive or disciplinary based upon the anticipated outcomes such as whether feelings of shame or pain resulted. The reliance on such outcomes to distinguish between punishment and discipline is problematic for several reasons. First, using outcomes of the method as the distinguishing feature means that the same behaviour could be interpreted differently across individuals and situations based upon the effects of the action. For example, the same exercise applied as punishment may have differential effects on the athlete depending upon physical fitness level. Further, punishment is not recommended because it teaches young people that adults have control over them, which also denies youth athletes the opportunities to learn important life skills such as self-control, problem-solving and independent thinking. Finally, a reliance on the outcomes of the methods precludes a preventative approach to harm of young people in sport [17]. Given the well-documented negative consequences for youth associated with punishment use [19], these methods should be avoided.

The inability of critical socializing agents in sport such as parents and coaches to distinguish between punishment and discipline is important for several reasons. The lack of clarity between punishment and discipline may limit stakeholders’ understanding of appropriate versus inappropriate behaviours, and without this understanding, harmful practices may be normalized and perpetuated. Further, the mechanisms of punishment, including the use of fear and control, may contribute to sport experiences for youth that are characterized by a lack of enjoyment and disinclination to continue sport participation [19,47]. The use of controlling practices such as punishment may also diminish opportunities for young people to learn important life skills such as problem-solving and teamwork through sport. Overall, we speculate that the advancement of developmentally appropriate disciplinary strategies is hindered by interpretations that normalize and promote the use of punishment in sport.

## 5. Limitations and Future Directions

The findings in the current study provide insight regarding sport stakeholders’ understandings of behaviour management strategies used in youth sport as well as their interpretations of these practices as disciplinary and/or punitive in nature. This research may stimulate questioning among sport stakeholders regarding the use of punishment and more developmentally appropriate disciplinary practices in sport. Nonetheless, the findings must be interpreted within the context of the current sample, which consisted of stakeholders affiliated with the same competitive boy’s baseball team in a specific geographic region. The fact that the participants came from the same team and had similar experiences may have limited heterogenous responses.

The stakeholders’ inability to distinguish between punishment and discipline indicates a need for education pertaining to developmentally appropriate behaviour management strategies. This need for education is heightened by the finding that the adults in the study often held positive views of punishment—an alarming interpretation given the plethora of evidence indicating the harmful effects of punishment [19,20,21,22,23]. The continued use of punishment in sport also speaks to the need for more research and practice on ways to better align the normative practices of sport with those used in other youth-populated domains. 

Several other areas of interest emerge for future research, including whether interpretations of punishment and discipline differ according to sport type, sport level, and identity variables such as gender, educational background, and years of coaching. Investigating the experiences of behaviour management among athletes with varying intersections of identity may expose alternative punitive methods or introduce more humanistic methods of discipline. Moreover, research exploring stakeholders’ perspectives of behavioural management strategies across different competitive levels could disclose an intensification of the utilization and endorsement of punitive measures under circumstances where there exists an amplified pressure to win.

## 6. Conclusions

All the athletes, coaches, and parents agreed that exercise, benching, and yelling are the most common behavioural management strategies used in youth sport. While the participants commonly interpreted yelling as punishment, they struggled to differentiate exercise and benching as punitive or disciplinary in nature. For many participants, punishment and discipline were perceived interchangeably based upon the assumption that both approaches achieve the same outcome of behaviour change. For participants who perceived a distinction between punishment and discipline, their interpretations were often characterized by a rudimentary understanding of punishment as negative and discipline as positive. This research extends current youth sport literature by exploring sport stakeholders’ understanding of common behaviour management strategies used in youth sport as being punitive or disciplinary in nature. Overall, the findings highlight the need to inform the sport community about the importance of using more developmentally appropriate strategies to foster positive sport experiences for youth athletes.

## Data Availability

The data that support the findings of this study are available from the corresponding author, upon reasonable request.
